# "Pee-in-a-Pot": acceptability and uptake of on-site chlamydia screening in a student population in the Republic of Ireland

**DOI:** 10.1186/1471-2334-10-325

**Published:** 2010-11-11

**Authors:** Deirdre Vaughan, Emer O'Connell, Martin Cormican, Ruairi Brugha, Colette Faherty, Myles Balfe, Diarmuid O'Donovan

**Affiliations:** 1 Project nurse/research health advisor, National University of Ireland Galway, Galway, Ireland; 2 Consultant in Public Health Medicine, Department of Public Health, Health Services Executive Dublin/Mid-Leinster, Tullamore, Ireland; 3 Professor of Bacteriology, Medical School, National University of Ireland Galway, Galway, Ireland; 4 Head of Department of Epidemiology, Royal College of Surgeons in Ireland, Dublin, Ireland; 5 Acting Chief Medical Scientist, Department of Virology, University Hospital Galway, Galway, Ireland; 6 Researcher, Department of Epidemiology, Royal College of Surgeons in Ireland, Dublin, Ireland; 7 Senior Lecturer in Social and Preventative Medicine, National University of Ireland Galway, Galway, Ireland

## Abstract

**Background:**

The aim of the study was to explore the acceptability and uptake of on-campus screening using a youth friendly approach in two Third Level higher education institutions (HEIs). This study is part of wider research exploring the optimal setting for chlamydia screening in Ireland.

**Methods:**

Male and female students were given the opportunity to take a free anonymous test for chlamydia during a one week programme of "pee-in-a-pot" days at two HEI campuses in the West of Ireland. The study was set up after extensive consultation with the two HEIs and advertised on the two campuses using a variety of media in the two weeks preceding the screening days. Screening involved the provision and distribution of testing packs at communal areas and in toilet facilities. In Ireland, chlamydia notifications are highest amongst 20-29 year olds and hence the screening criterion was aimed at 18-29 year olds. Urine samples were tested using a nucleic acid amplification test (NAAT). Following the screening days, qualitative in-depth interviews were conducted with participants about their experiences of the event.

**Results:**

Out of 1,249 test kits distributed in two HEIs, 592 specimens were collected giving a return rate of 47.5%. Tests excluded (54) were due to labelling errors or ineligibility of participants' age. Two thirds of those tested were females and the mean age was 21 years. Overall,3.9% (21/538) of participants tested positive, 5% (17/336) among females and 2% (4/191) among males. Participant interviews identified factors which enhanced student participation such as anonymity, convenience, accessibility of testing, and the informal and non-medical approach to testing.

**Conclusions:**

Screening for chlamydia using on-campus "pee-in-a-pot" days is an acceptable strategy in this population. This model can detect and treat asymptomatic cases of chlamydia and avoid many of the barriers associated with testing for sexually transmitted infections (STIs) in clinical settings.

## Background

Chlamydia is the most prevalent bacterial sexually transmitted infection (STI) in the western world [1]. In Ireland, the number of chlamydia notifications increased from 245 in 1995 to 6290 in 2008 [2]. While this may reflect a real rise in the burden of chlamydia infection, it also reflects an increase in provider awareness in chlamydia testing, test performance [3] and the introduction of laboratory notifications in Ireland in 2004. The real burden (numbers of infection) is likely to be higher than reported as chlamydia is asymptomatic in approximately 70% of women and 50% of men and thus may remain undiagnosed [4]. Prevalence studies [5,6] in young Irish people (including students) have shown similar rates of infection to the UK and else where in Europe [7-9].

In view of the asymptomatic nature of chlamydia, especially in women, there is recognition [10-15] that it is important to screen sexually active women aged less than 25 years [16]. Two approaches are proposed: (a) systematic, where all eligible young persons are systematically invited for screening, which requires the availability of a unique identifier for each individual to ensure all eligible persons are invited and not invited again except where indicated; and (b) opportunistic, where eligible young persons that are visiting/utilising clinical and non-clinical settings are invited to take a test.

Opportunistic screening in clinical settings has limitations [17] principally that it only reaches young people who are already attending health care settings [18]. In particular, men's underutilisation of primary care services means they may have few encounters with health care professionals for screening offers [19-21]. This is problematic as young adults are the highest risk group for acquiring chlamydia infection [22,23]. Balfe *et al*. reported that while young people expressed willingness to take up offers of opportunistic screening, they had major reservations about being offered screening by the family doctor and practice staff ('familiar strangers') because of concerns about anonymity [24]. Other recognised barriers for young people accessing sexual health services for testing include cost and inconvenience, and the fear and stigma commonly attached to STI testing [25]. Opportunistic screening also relies on heath care providers remembering to offer the test [26].

These issues highlight the need for additional strategies for screening that are free at the point of testing, easy to access, private and available in a variety of settings [27-29]. Screening strategies need to be 'youth friendly' and available outside of traditional medical facilities. Previous innovative approaches have ranged from screening in mobile vans to home testing, collecting samples in parks and in schools, screening on admission to residential settings such as detention facilities, job training programmes and the military [30-33]. Screening locations, which have a 'captive population' - schools, universities and correctional facilities, have been used especially to reach men [34,35]. While Lorimor *et al*. found young people especially men are willing to participate in non-clinical screening [36]. Balfe *et al*. found that screening needs to offer privacy from 'a public audience' with confidential testing [37].

The purpose of this study was to test the acceptability and uptake of on-campus chlamydia screening in two Irish HEI settings using a youth-friendly approach. Young people in HEIs are a group of sexually active persons who are at high risk of contracting STIs [38]. HEIs are potentially important settings for STI screening in most countries as they provide access to a large population of young people. In Ireland the HEI student population represents over 10% of the total population [39].

This study was part of wider research exploring the optimal setting for chlamydia screening in Ireland which also included a national pilot study. Ethical approval for this study was granted by the Irish College of General Practitioners and the Research Ethics Committee of the National University of Ireland Galway, Ireland.

## Methods

A sequential explanatory mixed method study design that combines both quantitative and qualitative research methods was used [40]. In mixed methods research designs, researchers use both quantitative and qualitative methods and data in the same study or series of studies; in the sequential explanatory approach, qualitative findings are used to explain and understand quantitative findings [41]. Using both qualitative and quantitative data in a study can produce more complete knowledge needed to inform clinical practice [41]. The quantitative data comprised of screening data results and qualitative findings were from semi-structured interviews conducted with students who took part in the event.

### On-site screening

#### Study population

The study population was male and female students attending two HEIs, a university and an institute of technology, (student populations of 16,470 and 4,973) in the West of Ireland. Irish HEIs include universities, colleges and institutes of technology. (Higher education is the education level following the completion of a school providing a secondary education such as high school or a secondary school).

The total student population (full time and part time) in the university consisted of 5,213 males and 7,336 females with 2,794 males and 2,026 females in the institute of technology. The highest attendants in both settings were between the ages of 19-21 years [42]. Students from non-manual, skilled-manual, semi-skilled and unskilled backgrounds are better represented in the institute of technology with 32.4% compared to 26.3% in the university sector [42].

Internationally, the highest rates of chlamydia are documented as occurring in young people aged less than 25 years [43]. In Ireland, the highest rates of notified cases are among those aged 20-29 years [44]. For the purpose of the study, screening was aimed at men and women aged 18-29 years.

#### Planning of screening programme

Several planning meetings were held with staff from the student health units and student unions to plan logistics and screening protocols (including treatment and follow-up) for a publicised mass screening programme, to be implemented during a one week 'sexual health awareness and guidance' (SHAG) week, which is an annual student event designed to promote positive sexual health. Colourful posters and information leaflets attracted attention to the program and were distributed around campuses. Media releases, radio broadcasts, email alerts and newspaper articles were used to publicise the event.

#### Testing packs

Testing packs (small specimen bags containing a 25 ml urine container, a pen and an information card) were designed for the study. Testing was anonymous and each pack was identified through a unique code. Participants who chose to take a test were instructed to read the information card, write their mobile number and date of birth on the urine container, urinate in this container and place it in the specimen bag. Information leaflets on chlamydia testing were readily available and distributed to participants which covered the following: information on the research project including age criterion, what is chlamydia and how it is transmitted and diagnosed, what happens if the test is positive and information on telling your previous/current partners. Additional information on local and national sexual health services was also supplied. Consent for testing was implied through the participation and provision of personal identifying details on the sample container. Specimen collection boxes were located inside toilet areas where participants were instructed to drop their specimen bag. Testing packs were made available for three to four hours each day during the one week screening programme.

Project researchers collected the specimen bags and transported them to the laboratory services each day where the urine specimens were frozen and batch tested with Polymerase Chain Reaction (PCR) testing technology. The test used was the *COBAS*^® ^*TaqMan*^® ^*CT Test v2.0 *manufactured by Roche Diagnostics, Switzerland. The sensitivity and specificity of this test *(as per the Roche Cobas Taqman CT test, v2.0 Preparation kit) *is reported as 95.7% and 99.8%. With a prevalence rate of 3.9%, the positive predictive value of this test in our study population is 95.1%.

#### Peer volunteers

Student (peer) volunteers were recruited to distribute testing packs and information leaflets to potential participants and around the student campuses. The 35 volunteers were given an educational session on chlamydia and the background to the research project. Volunteers were given incentives (€25 voucher) for their participation (approximately four hours each). Volunteers were dressed informally and were easily identified through colourful t-shirts with "Pee-in-a-Pot Volunteer" printed on the back.

#### Screening approach

The approach used in the study was adapted during the one week to maximise privacy for participants. While testing packs were initially distributed in communal areas: as the event progressed male and female toilet areas became the focal point for testing. Packs were distributed and made available around sinks, mirrors and inside toilet cubicles, which led to more participants self-selecting for screening. The most popular approach was for students to pick up a pack themselves in the toilet cubicle. Male volunteers were allocated to male toilets and male orientated entertainment venues, such as snooker rooms while female volunteers were allocated to female toilets and other communal areas.

#### Notification of results

Participants with negative results for chlamydia were informed by way of a carefully worded standard SMS text message (*your recent test result was negative*). Participants with a positive result were telephoned by a medical doctor and invited to attend for an appointment at their respective student health units. Positive cases were treated with 1 g of Azithromycin free of charge and given a study information leaflet about their result. Positive cases were asked by a medical doctor for their consent to be referred to the project nurse/health research advisor for partner notification and follow-up. This project nurse/health research advisor discussed partner notification, further STI testing and sexual health education by phone anonymously. Participants were then contacted 3-6 months after treatment for a retest.

### Data analysis

Chlamydia results, date of birth, mobile phone number, outcomes for partner notification, STI testing and retest results were entered on to a secure Microsoft ACCESS database. Analysis was conducted through Statistical Package for the Social Sciences (SPSS version 17). Cross tabulation and descriptive frequencies between key variables were carried out.

#### Semi-structured interviews with participants

Following the one week screening event, participants in both campuses were invited for an interview to explore their views and experience of the event. A mass recruitment email with an information sheet was sent via the student unions intranet system inviting students who had participated in the "pee-in-a-pot" for an interview. All registered students in both campuses would have received this mass email (HEI 1-16,470 and HEI 2 - 4,873) Potential participants were instructed to text 'YES' to a mobile phone number if they wanted to be interviewed. Project researchers conducted interviews by telephone after receipt of verbal consent. Participants who tested positive for chlamydia were also invited for an interview during their follow-up consultations. Interviews were anonymous and a gift voucher (€30) was given to all interviewees.

A small number (7) of qualitative semi-structured interviews were conducted (6 female, 1 male) by two project researchers using a topic guide. Data saturation was reached quickly. Transcripts were coded to identify emerging themes and checked by two project researchers. Thematic analysis was used to generate emerging themes.

## Results

### Screening

Three and a half screening days were conducted in one HEI (HEI 1) and three screening days were held in the second (HEI 2). The latter campus is a more widely distributed campus with several buildings located in different areas. A total of 538 tests were eligible for inclusion in the study. A total of 1249 testing packs was distributed across the two campuses, of which 592 (47.5%) were returned with specimens. Forty five tests (all of which were negative) were received from participants outside the target age range for the study (18-29 years) and were excluded from the final analysis. Nine further tests, which were excluded because of specimen labelling errors or inadequate personal identifying information were not analyzed.

#### Results of screening

Three hundred and thirty six (63%) of the study samples were from women and 191 (35.5%) were male. The sex of the participants was not given in eleven samples. The mean age of participants was 21 years. The uptake rate in HEI 1 was 37.2% (183/493) and in HEI 2 was 54.2% (409/756).

The overall chlamydia positivity rate amongst participants with test results was 3.9% (21/538) with 5% positive (17/336) in females and 2% (4/191) in males. The highest rate in both age groups was amongst the 20-24 years (Table 1). Although differences are found in positivity rates and uptake between the HEIs, these are generally not statistically significant (see Table 1 and 2).

**Table 1:** Positivity rate by sex and age group

	Female		Male		
	CT Pos (%)	95% CI	CT Pos (%)	95% CI	P value*
**Age (years)**					
18-19	4/93 (4.3%)	0.18,8.42	1/56 (1.7%)	0,5.26	0.687
20-24	13/214 (6%)	2.87,9.27	3/112 (2.6%)	0,5.67	0.197
25-29	0/29		0/23		
					
**Total**	**17/336 (5%)**	**2.72,7.4**	**4/191 (2%)**	**0.06,4.12**	**0.1086**

* P values calculated using Fishers exact test

**Table 2:** Test results of participants and positivity rates per setting

	**HEI 1**.			**HEI 2**.			
	Total	CT Pos(%)	95% CI	Total	CT Pos(%)	95% CI	P value*
**Age (years)**							
18-19	41	2 (4.9%)	0,11.47	109	3 (2.8%)	0,5.82	0.61
20-24	105	8 (7.6%)	2.55,12.69	230	8 (3.5%)	1.11,5.85	0.106
25-29	21	0		32	0		
							
**Sex**							
Male	76	3 (3.9%)	0,8.33	115	1 (0.9%)	0,2.57	0.30
Female	82	7 (8.5%)	2.49,14.59	254	10 (3.9%)	1.55,6.33	0.14
Unknown	9			2			
							
**Total**	**167**	**10 (5.9%)**	**2.39,9.59**	**371**	**11 (2.9%)**	**1.24,4.68**	**0.146**

*P values calculated using Fisher exact test

### Case Management

Out of the twenty one positive cases, eighteen participants were treated for chlamydia. Three participants with a positive result were not contactable. Reasons for this included two incorrect mobile phone numbers and one mobile phone was disconnected. The mean waiting time for receiving test result was 4.5 weeks. Fifteen positive cases were treated at the student health units, two were treated at their respective family doctors and one positive case was treated at a genitourinary (GUM) clinic in another country.

Partner notification was conducted with fifteen participants - ten by the project nurse/research health advisor and five by a practice nurse from one of the HEIs. Consent for referral to the project nurse/research health advisor was not given by one participant and two refused to discuss partner notification. Patient referrals were the preferred options with thirteen of the positive cases notifying their previous and current partners themselves. Four provider referrals were conducted by the project nurse/research health advisor.

All participants who tested positive were advised to attend for further STI screening and appointments were made for seven participants to attend the local GUM clinic. In total, three participants went on to have further STI screening and eleven participants did not attend for further screening. No additional STIs were detected.

### Interviews with participants

Themes that emerged during the interviews are identified under the sub-headings below. Respondents' reactions to the on-campus screening programme were positive overall, 'feeling it was a good experience'. Three interviewees commented that they would take part in such an event again

.. I thought it was a good idea because all the students were on campus and I thought it was very easy to use. (Interview 6. female)

#### Barriers to testing in clinical setting

Participants discussed factors which would deter young people from testing in clinical settings. These factors included embarrassment, difficulties talking about sexual health and lack of knowledge about STIs. Attending a clinical setting was perceived to be 'more public' through having to sit in waiting rooms.

#### Testing in non-clinical setting: Accessibility and convenience of testing

All of the participants spoke very enthusiastically about the convenience of having testing packs readily available on-campus, especially in toilet facilities. 'Hassle-free', no fuss', 'handy' and 'easy to do' were frequent comments made by different interviewees. The 'non-technical' nature of urine testing was appreciated by many and the fact participants could 'do the test themselves'.

Well because it was convenient, it was just on campus, it was something you didn't have to like go a specific place and the way it was handled like. (Interview 2. female)

#### 'No names and mobile phones'

Respondents stressed the importance of the testing being anonymous with interviewees feeling it was clearly linked to the successful uptake. Testing was perceived to be 'a private issue' for participants. Most felt that they would not have participated if they had to give their full names. The level of personal information required (i.e. mobile phone number and date of birth) for the event was deemed acceptable by all of the interviewees.

No names, that was a bit I liked most, my number just seems fine but I would hate to put down my name. (Interview 2. female)

#### Less embarrassment

The 'usual' embarrassment and shame associated with STI testing was perceived to be considerably reduced by the event. Several participants stated they experienced little-to-no embarrassment and shame because 'everyone was doing it' (the test). However, one participant commented that having peer volunteers distributing testing packs in the bathroom may have caused embarrassment to some students.

I didn't think much of it; I know that some people were a little bit embarrassed because there would have been a queue into the bathroom while they were offering it. (Interview 3. female)

However, as the event progressed and where logistically possible, testing packs were provided in toilet cubicles,

I think a lot of people would have just been like, oh well it's in the cubicle with me, the box is right there, there's nothing stopping me, I'd be stupid not to do it like, you know. (Interview 1. female)

#### Peace of mind

A common theme which emerged among respondents was the positive impact of taking the test. Testing was seen to give 'peace of mind' and 'a sense of relief'. Half of interviewees stated they felt pleased with themselves because they felt they had taken a positive step towards looking after their health.

....and you kind of have piece of mind because you feel better about yourself because you feel that you're looking after yourself even though you didn't actually get to the doctor (Interview 1. female)

#### Non-medical nature of setting

The informal and casual approach to testing was raised by many and contributed to the high participation rates. Respondents enjoyed the 'light hearted' approach to testing. Many commented on the use of the user 'friendly' language (such as pee-in-a-pot) and the 'openness' of displaying testing materials along with the presence of peer volunteers/project staff wearing the colourful t-shirts. All of these generated an atmosphere of fun, taking the 'fear factor' out of testing for many.

Yeah and the whole approach to it wasn't like eerie you know, scary like. The way the name, "Pee in a Pot" like, it's catchy; it's a bit more light-hearted than if you were to actually to go to a proper clinic you know. (Interview 4. male)

Contextualising the event and holding it during the sexual health awareness week was important for two of the interviewees as they believed students were 'in that frame of mind'.

....it was during SHAG week so there was a big emphasis on like safe sex and contraception and it just made people really, really aware of what can happen. (Interview 7. female)

Half of the respondents commented on the positive role played by the student union and student health services in the event as these actors were viewed as 'trusted sources'.

#### Use of peer volunteers

Interviewees felt that it was 'a good idea' to use peers in the event. Age was important with most participants preferring to be approached by someone 'young like themselves'. Perceptions that older persons have more traditional views of sexual health were expressed while the use of young people brought a more open and 'modern' outlook to the event.

Because they're more, the older nurses would be like more old fashioned and traditional I think, they'd be like; oh you shouldn't be doing something like that outside of marriage.......and like the younger nurse would be more modern and understanding, you'd be more comfortable with a younger person.... (Interview 7. female)

### Test Results

Participants commented on the convenience of text messages for negative results and were pleased with the standard text that received which they felt protected their privacy (chlamydia was not mentioned in the text).

It didn't say what it was. Like the way the text message was really good because only I would know what, it didn't say, it just said from a recent test or something like that but it didn't actually explicitly say you know, it was done in a good way. (Interview 2. female)

Several participants commented on the delay for results with two participants commented that they were worried when they did not hear.

### Recommendations for future testing

When asked about improvements for future testing, most suggested that all testing kits and drop off boxes could be located inside the toilet cubicle to allow for complete privacy. Several interviewees felt the urine collection drop off boxes should be secure for any future event.

I suppose putting the stuff inside the cubicle maybe because then no one sees if you're taking it or not. I think more so for girls than boys. (Interview 3. female)

## Discussion

This study showed on-campus screening for chlamydia generated high uptake and was acceptable to students. The discreetness of testing, where students could take an anonymous chlamydia test in a toilet cubicle, suggests a particular strength of the screening programme design which ensured confidential testing. The overall rate of infection of 3.9% in students was comparable to that in other European studies [45-47]. The positivity rate of 5% was found in females and is similar to that reported by O'Connell *et al*. which found a rate of 4.8% within a similar population attending student heath units in the Republic of Ireland [48,49].

The rate (2%) found in males is broadly comparable to European studies [30] and is slightly lower than UK rates [50]. Positivity rates were generally higher in HEI 1 than in HEI 2, among females 8.5% (7/82) compared to 3.9% (10/254) and among males 3.9% (3/76) compared to 0.9% (1/115). However, differences were based on small numbers, precluding inferences to the larger populations.

### Gender difference in uptake

Overall male participation in the event was lower (191) than female (336). The lower number of males taking up testing in the testing, although no statistically significant suggest that males may distance themselves from chlamydia by labelling it as a 'women's disease' as has been reported [51]. Others have commented that males are less likely to accept screening than females [52,53]. Possible reasons may be that males are less knowledgeable about chlamydia than females of the same age [54,55]; and males may become more embarrassed, anxious and ashamed about being asked to take part in chlamydia screening programmes [56]. Given the nature of male toilet facilities where there are fewer private cubicles, men may have felt more inhibited about taking the test privately in a toilet cubicle or might not have wanted to take the time.

There were also differences between the settings in the proportions of males and females who accepted the screening offer, being almost equal in HEI 1 (75:73), while in HEI 2 there were nearly twice as many women as men (244:114). Reasons may include: there were more male peer volunteers circulating in the HEI 1 campus who were very pro-active and enthusiastic about the event, interacting more with potential male participants. Male volunteers in the HEI 1 were also in the toilets areas distributing tests kits more frequently. Some of the male volunteers were representatives of the student union and may have been known to many of the potential participants. As one interviewee commented during the interview, the HEI 1 Student Union is regarded as a "trusted source" by students.

The proportion (35.5%) of males in the event compares favourably to a UK chlamydia screening postal study [57] which showed a lower participation rate (18.9%) of those males offered screening. While male participation is encouraging given the evidence young males are often identified as a difficult population to reach [58-60] and have different health seeking behaviour than females [61], an Australian study found cash incentives encouraged male participation in screening [62].

### Screening model

The screening approach used and developed in this study demonstrates several advantages. Narratives in the interviews show the importance of normalising testing. The 'openness' and 'lighted hearted, fun' approach removed the fear factor often associated with STI testing and helped to normalise testing. Participants welcomed the 'informal and non-medical' approach to testing. The 'non-technical' nature of urine testing was 'easy to do' and participants appreciated 'doing the test themselves'. The psychosocial impact of testing was commented on in the narratives with testing giving more than half of the participants 'peace of mind' and feeling good about 'taking responsibility for their health'. This is consistent with other studies [63] which suggest testing for chlamydia has the effect of relieving rather than creating anxiety [64].

Participant's emphasised the importance of accessibility and convenience during the interviews, which is consistent with others' findings: young people are less likely to accept screening if they believe that screening will inconvenience them [65-67]. Privacy and anonymity was also crucial to uptake. This model provides a successful framework which offers convenient and accessible testing which ensures privacy and confidentiality for participants. The model developed and tested in these two HEIs now needs to be refined before replication. As raised in the interviews, some participants may have been embarrassed by testing kits been handed out in toilet areas. To further enhance privacy and minimise social embarrassment, the model could be tailored to locate all testing packs and collection boxes within toilet cubicles as recommended by several interviewees.

This approach may prove to be a useful model to use in a wider variety of non-clinical community settings. Community based settings for early school leavers, probation and homeless services could be explored as well as non-educational settings such as employment occupational health settings. This model does not seek to replace the need for clinical based screening but rather utilises opportunities to reach target populations who use clinical settings infrequently or who might be reluctant to accept STI screening offers in such settings. This model may also prove to be considerably less expensive than screening in clinical settings, and a paper presenting a cost effectiveness analysis of this approach is in preparation. While the UK National Chlamydia Screening Programme (NSCP) has a portfolio of approaches to screening, it also encourages screening opportunities in commercial pharmacies, universities, colleges, and other venues. Engaging other venues can maximise access and opportunities to reach target populations especially the more difficult to reach young people [68].

The study had some limitations. While the use of peer volunteers appeared to generate an 'informal and light hearted' approach, the full impact of their involvement was not explored.

Numbers tested were small, based on only two HEIs and might not be representative of the total population of students in HEIs.

Despite all registered students in both campuses receiving the mass recruitment email inviting participants for an anonymous interview, the respond rate was low with only 7 interviews being conducted. It is uncertain as to the low number of respondents.

## Conclusions

This study demonstrated that on-campus self-selected screening and confidential testing is feasible and acceptable to students (during SHAG week when participants were open to and used to seeing information and guidance on sexual health). The benefit of using this strategy is that it can detect asymptomatic cases of chlamydia in these young people. It eliminates many of the barriers associated with STI testing in clinical settings because it is easy to access, convenient, confidential and private. It helps to promote chlamydia testing as "responsible behaviour" and it normalises testing [69].

The study contributes to the growing body of evidence around the effectiveness of screening approaches in non-clinical settings and helps to identify gaps identified in the literature [70]. Third level education institutions (HEIs) provide an easily accessible population, where screening is acceptable if discretely available.

## Competing interests

The authors declare that they have no competing interests.

## Authors' contributions

DV designed screening event and materials used, managed and participated in the screening events, recruited peer volunteers, collected and analysed data and drafted manuscript. EOC contributed to study design, participated in study event, analysed data and drafted manuscript. DOD contributed to study design and coordination, data analysis and helped draft the manuscript. CF and MC participated in the design, monitored and guided laboratory procedures. RB and MB assisted in study design, data analysis and drafting of manuscript. All authors have read and approved the final manuscript.

## Pre-publication history

The pre-publication history for this paper can be accessed here:

http://www.biomedcentral.com/1471-2334/10/325/prepub
